# Mechanical behavior of a hydrated perfluorosulfonic acid membrane at meso and nano scales†

**DOI:** 10.1039/c9ra00745h

**Published:** 2019-03-26

**Authors:** Cong Feng, Yan Li, Kunnan Qu, Zhiming Zhang, Pengfei He

**Affiliations:** College of Materials Science and Engineering, Shanghai Key Lab of Metal Functional Materials, Tongji University Shanghai 201804 China; School of Automotive Studies, Tongji University Shanghai 201804 China; School of Aerospace Engineering and Applied Mechanics, Tongji University Shanghai 200092 China ph232@tongji.edu.cn

## Abstract

Perfluorosulfonic acid (PFSA) is widely used as the membrane material for proton-exchange membrane fuel cells, and its mechanical properties directly affect the stability and the life of the internal structure of the proton exchange membrane. In the present research, mechanical behaviors of hydrated membranes are investigated through macro-mechanical experiments and nano-simulation. The commercial products of Nafion® 117 and Nafion® 212 are used as tensile testing samples, and the latter manifests a larger swelling ratio, smaller linear-elastic region, and higher Young's modulus at the same humidity. It is found that in comparison to Nafion 117, humidity (especially at less than 9 wt%) yields profound effects on true stress–true strain curves of Nafion 212. Further, two types of PFSA ionomers, representing the nanoscale parts of Nafion and Aquivion membranes, respectively, are studied on the uniaxial tensile deformation through molecular dynamics simulation, and the effects of side chain, water content, backbone length, and strain rate are examined. It is noticed that long side chains decrease the winding degree of polymer chains for the PFSA ionomers with the same backbone, and pinhole failure causes the declining trend of stress–strain curves. For meso- and nano-scale PFSA polymers, the difference between the volume of uptake water and swelling volume can be used to roughly judge the aggregation degree of polymer chains and explain the elastic–plastic deformation mechanism. The results of this work will serve as a baseline for understanding the swelling and mechanical behavior of PFSA membranes.

## Abstract 2

Perfluorosulfonic acid (PFSA) is widely used as the membrane material for proton-exchange membrane fuel cells, and its mechanical properties directly affect the stability and the life of the internal structure of the proton exchange membrane.

## Graphical abstract

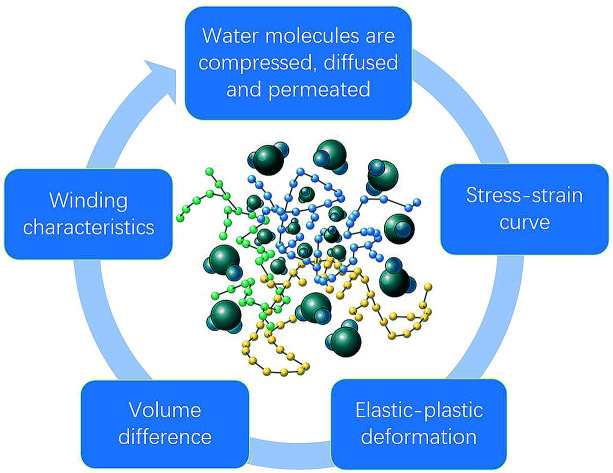

## 1. Introduction

Polymer electrolyte membranes are widely used in fuel cells, batteries, and solar cells due to their mechanically and chemically stable forms of electrolyte. These materials possess covalently bonded ionizable groups, which greatly influence the mechanical behavior of proton-exchange membrane fuel cells (PEMFCs). Currently, the durability of membranes during long-term cyclic operation remains a major obstacle for the widespread commercialization of PEMFCs. Polymer electrolyte membranes are prone to chemical degradation and mechanical damage,^1–3^ and mechanical stresses in membranes due to hygro-thermal cycling are considered to be the main factor for mechanical damage.^4–6^ Hence, the mechanical behavior of polymer electrolyte membranes and its dependence on hydration is of great research interest for designing robust PEMFCs.

Perfluorosulfonic acid (PFSA) membranes, such as Nafion and Aquivion, are the most commercially used membranes. Multifarious experimental studies, including dynamic mechanical method, monotonic tensile, stress relaxation and creep, have demonstrated that elastic modulus, yield stress, and post-yield behavior of membranes are strongly dependent on strain rate, temperature, and humidity.^7–16^ It is reported that elastic modulus and yield stress significantly decrease with the increasing temperatures and humidity and at the same time, increase with the increasing strain rates.^17,18^ Mechanical loading generally causes the internal deformation of membranes including the orientation of polymer chains, crystallization, viscoelastic relaxation, cavitation, and rupture. Therefore, the macro mechanical responses are more related to structural deformation at nanoscale. Molecular dynamics (MD) simulation is a powerful tool to detect these changes at the atomic level.^19,20^ In the current research realm, MD simulation has successfully applied to study the physicochemical properties of polymers, including structural, transport, and mechanical properties.^21–27^

Some earlier studies^28–33^ have reported the applications of MD simulation on PFSA membranes. Allahyarov and Taylor investigated the effects of stretching-induced structure orientation on proton conductivity and revealed that uniaxial stretching caused the elongation of hydrophilic regions along the stretching direction.^28^ Daly *et al.* studied the shear viscosities of Nafion oligomers under a low-solvent volume fraction and expressed that their viscosities depended on hydration and counterion type.^29^ Ozmaian and Naghdabadi^30^ investigated the physical properties of Nafion under uniaxially loading at a wide range of temperatures and demonstrated that the increase in temperature reduced the values of yield stress as well as the orientation of polymer chains increased the glass transition temperature and enhanced the self-diffusion coefficient of water in hydrated Nafion. Abu-Hakmeh *et al.*^31^ examined the effects of uniaxial deformation on structure and transport properties of hydrated Nafion 117 membrane. It was found that the sulfonate distribution and the sulfonate–hydronium correlation became stronger through the deformation, whereas the hydronium ion solvation and the internal structure of water were not dependent on the deformation.

Nafion membranes with thinner thickness manifest special characteristics, such as reduced swelling,^34,35^ lower ionic conductivity,^36^ and higher modulus.^37–39^ Although these characteristics are affected by thickness scales, they are actually more related to microstructure. Based on earlier experimental and theoretical findings, the current work aimed at exploring the mechanical behaviors of hydrated PFSA membrane, and analyzing the relationship between the swelling characteristics, stress–strain curves and deformation process for both meso- and nano-scale ionomers. The bulk films of commercial membranes (Nafion® 117 and 212) with different thicknesses were investigated by exerting tension along the length direction at different humidity. Two types of PFSA ionic polymers, representing nanoscale parts of Nafion and Aquivion membrane, respectively, were used to analyze their mechanical behaviors at various water contents through MD simulation.

## 2. Experiment

**Fig. 1:**
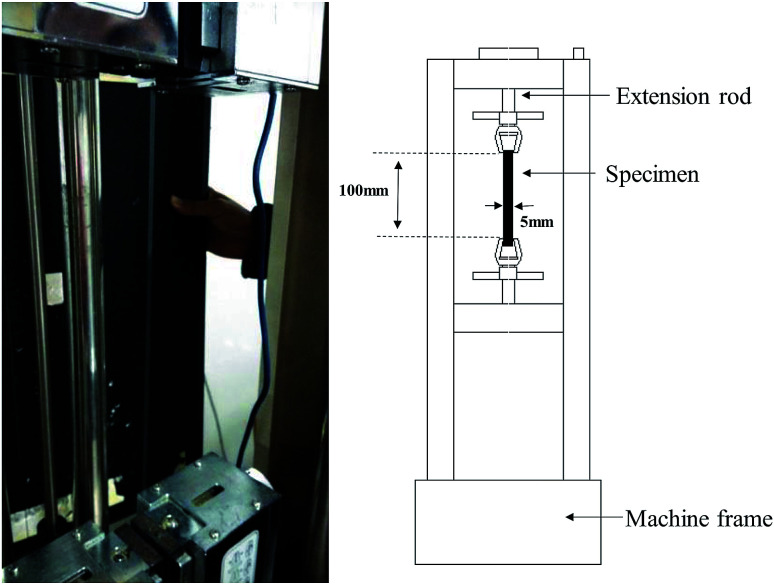
Pictorial representation and schematic diagram of double-wire rod electronic pull machine.

The tensile mechanical responses of Nafion® 117 (thickness ∼180 μm) and Nafion® 212 (thickness ∼51 μm) membranes were measured in a double-wire rod electronic pull machine at room temperature (Fig. 1). As Nafion® 212 has a much higher strength, a larger strain rate (5 min^−1^/0.08 s^−1^) was employed in comparison to Nafion® 117 (2 min^−1^/0.03 s^−1^).

The prepared samples for both Nafion® 117 and 212 had the same size (5 mm wide and 100 mm long). For each sample, width and thickness were measured with a caliper and a micrometer, respectively, at three locations along the sample before testing. The samples were first dried at 100 °C in a vacuum oven for 24 h. Among them, three were used as dry samples, and others were soaked in deionized water for different periods of time (1 m, 3 m, …, 1 h, 3 h,…, 24 h). This procedure is to acquire the hydrated membranes with various humidity. Then water was rapidly removed from the membrane surface to measure their sizes and weights. The average values were used. Based on these data, the corresponding swelling ratio (volume swelling) and humidity (water content) were calculated. In order to achieve the desired humidity conditions as well as to keep the samples stable during tensile testing, each sample was wrapped in a cling film in the whole tension process. The two clamped tension ends were left unwrapped. Because we only investigated a shorter elastic–plastic deformation process (true strain less than 0.7), the cling film were considered to have no effect on the tension results.

As the membrane were very thin and smooth, the clamped ends were pasted by 3 M adhesive tape to keep them fixed during stretching. Three samples with similar humidity were used for tensile testing. Before applying a force, the clamp was manually adjusted until the initial force applied to the specimen was less than 0.01 N, thus eliminating the initial slack caused by thermal and swelling expansions. The connected computer exported the values of applied forces and induced displacements during tensile testing.

## 3. Simulation

**Fig. 2:**
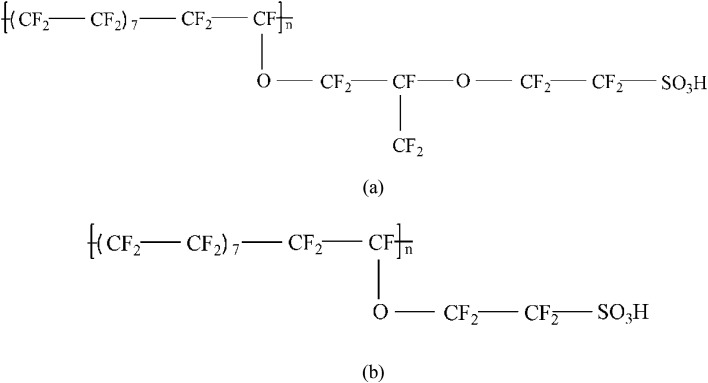
Chemical structures of (a) Nafion and (b) Aquivion membranes.

Two types of PFSA membranes with the same backbone and different side chain were simulated. They had the similar chemical structures of DuPont Nafion (EW ∼1100 g mol^−1^) and Solvay Aquivion (EW ∼910 g mol^−1^) membrane, as illustrated in Fig. 2a and b, respectively. PFSA membranes with EWs of 1144 and 978 at nanoscale were denoted as Nafion and Aquivion ionomers, respectively. These two PFSA ionomers had four different hydration levels (*λ* = 3, 6, 9, 14, where *λ* = H_2_O/HSO_3_), and each of them consisted of different chains (the number of chains was decided by backbone length (the number of monomer in a chain)) based on system size. In a wet environment, all of the oligomers were considered to be completely ionized, and hydronium ions (H_3_O^+^) were used as the counterions to sulfonate ions (SO_3_^−^) for the sake of electroneutrality.

The initial system configurations (density of 0.1 g cm^−3^) were generated by randomly arranging the molecules in a cubic box using Packmol software. The full-atomistic MD simulations were performed in large-scale atomic/molecular massively parallel simulator (LAMMPS)^40,41^ software developed by Sandia National Laboratory. A modified CHAMM force-field function^42^ was applied to describe the interaction between hydrated membrane atoms. The potential energy of the system comprised of Lennard-Jones potentials for van der Waals interactions (*E*_LJ_), electrostatic energy (*E*_coulomb_), bond stretching energy (*E*_bond_), angle bending energy (*E*_angle_), and dihedral angle torsional energy (*E*_dihedral_).

1 *E*_total_ = *E*_LJ_ + *E*_coulomb_ + *E*_bond_ + *E*_angle_ + *E*_dihedral_

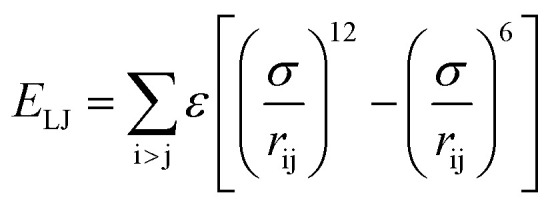

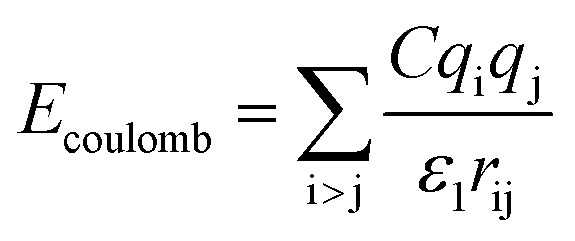

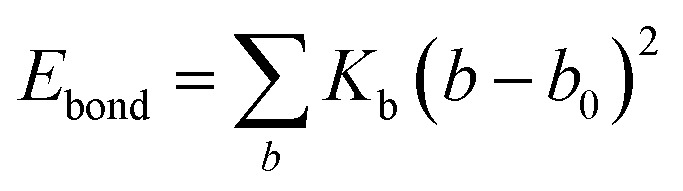

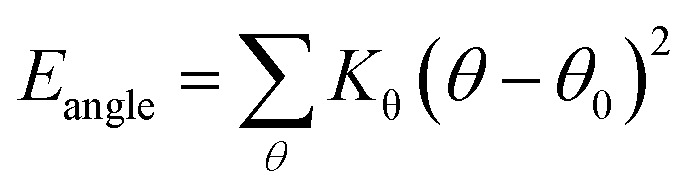

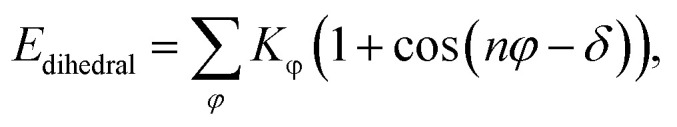
where *K*_b_, *K*_θ_, and *K*_φ_ signify bond, angle, and dihedral angle force constants, respectively; *b*_0_ and *θ*_0_ are the equilibrium values for bond length and bond angle, respectively; *ε* is the Lennard-Jones well depth; *σ* is the distance at the Lennard-Jones minimum; *q*_i_/*q*_j_ is the partial atomic charge; *ε*_1_ is the effective dielectric constant; and *r*_ij_ is the distance between atoms i and j.

The initial structures of amorphous PFSA ionomers were equilibrated using annealing procedure and relaxed by performing 20 anneal cycles in order to achieve the target densities (hydrated Nafion ranging from 1.70 g cm^−3^ to 1.85 g cm^−3^ and hydrated Aquivion ranging from 1.80 g cm^−3^ to 1.95 g cm^−3^). The isothermal-isobaric ensemble (NPT) MD simulation with a time step of 0.5 fs was adopted. The temperature of each cycle was gradually increased from an initial temperature (300 K) to a mid-cycle temperature (1000 K) and then again decreased to the initial temperature, and the total time of the anneal procedure was 10 ns.

Before stretching, an equilibrating NPT MD simulation was conducted for each model at *T* = 300 K and *P* = 1 bar for 20 ns with a time step of 1 fs. The equations of motion were integrated using Verlet algorithm.^43^ The Nose–Hoover thermostat^44^ with a damping relaxation time of 0.1 ps was employed to control temperature and pressure. The uniaxial deformation was then applied gradually at a rate of 10^9^/s in the *x*-direction of the above-obtained equilibrium structure.

## 4. Results and discussion

### 4.1 Macro-mechanical experiment of hydrated Nafion membranes

#### 4.1.1 Swelling behavior

**Fig. 3:**
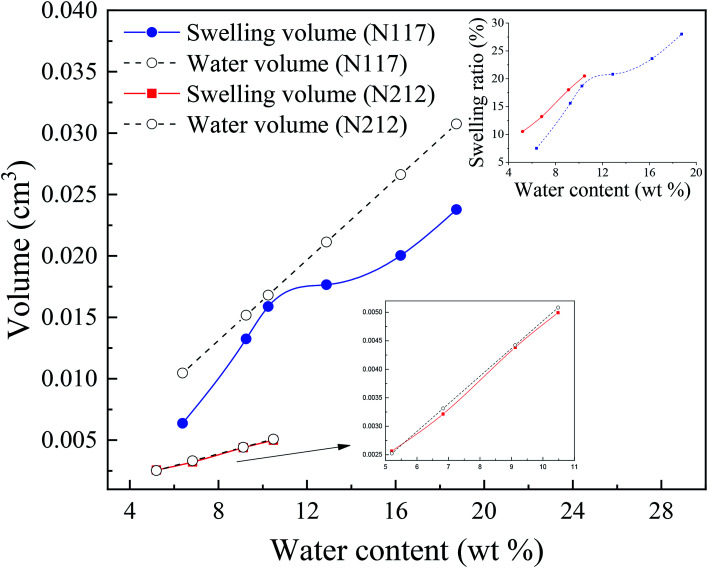
Volume-water content curve for Nafion® 117 and 212. The embedded figure in top right corner shows the relationship of swelling ratio and water content (blue color and red color are for Nafion® 117 and 212, respectively).

The swelling behavior significantly determines the mechanical responses of hydrated membranes. The swelling volume (*V*_s_), volume difference of Nafion after and before water absorption, was first investigated. It was found that the swelling volume was not consistent with the volume of uptake of water (divided the quality of water by its density (1.0 g cm^−3^)), with the latter larger than the former in both membranes at the same humidity, as shown in Fig. 3. It is known that the swelling phenomenon is occurred when water molecules permeate and diffuse into ionomer, weakening the interaction between ionomers and increasing the spacing between molecules, then causing the volume expand. Therefore, it can be inferred that Nafion® 117 and 212 exhibited the characteristics of entanglement of polymer chains, water molecules in the permeating and diffusion process were compressed. However, Nafion® 212 had much smaller difference between water volume and swelling volume (*V*_w–s_) than Nafion® 117; the former was two orders of magnitude lower than the latter (O (mm^3^)), indicating better intermolecular dispersion of polymers.

It is worth mentioning the soaking process of Nafion® 117. In the initial period, the volume difference of *V*_w–s_ decreased first and then increased, reaching the minimum at the humidity of 10.3 wt%, which was similar as the intersection in Fig. 3c for Nafion® 212. This demonstrated that the water introduction weakened the entanglement degree of polymer chains in the initial stage, then as water content increased, the entanglement was not more weakened and water molecules continued to be squeezed and diffused. These results indicated that the thickness and structure of Nafion® 212 was conducive to the diffusion of water molecules.

The inset figure displays the relationship between swelling ratio and water content of Nafion® 117 and 212 membranes. Nafion® 212 manifested higher swelling ratio as compared to Nafion 117; however, with the increase in soaking time, the swelling ratio of Nafion® 117 increased faster than that of Nafion 212 due to the former with a greater thickness. After 24 hours, water uptake and swelling ratio for both Nafion membranes reached steady values. Nafion 117 yielded the maximum (saturated) water uptake (18.75 wt%) and swelling ratio (28%), whereas the values for Nafion 212 were 10.48 wt% and 20.5%, respectively.^45,46^

#### 4.1.2 True stress–true strain curves

The true stress–true strain (TS–TS) relationship was used to analyze the mechanical deformation of hydrated Nafion membranes.*σ*_true_ = (1 + *ε*_e_)*σ*_e_.*ε*_true_ = ln(1 + *ε*_e_).where *ε*_e_ and *σ*_e_ are engineering strain and stress, respectively; *ε*_true_ and *σ*_true_ are true strain and true stress, respectively. In our study, the elastic–plastic deformation of Nafion membranes was investigated for *ε*_true_ less than 0.7 (*ε*_e_ = 1.0).

**Fig. 4:**
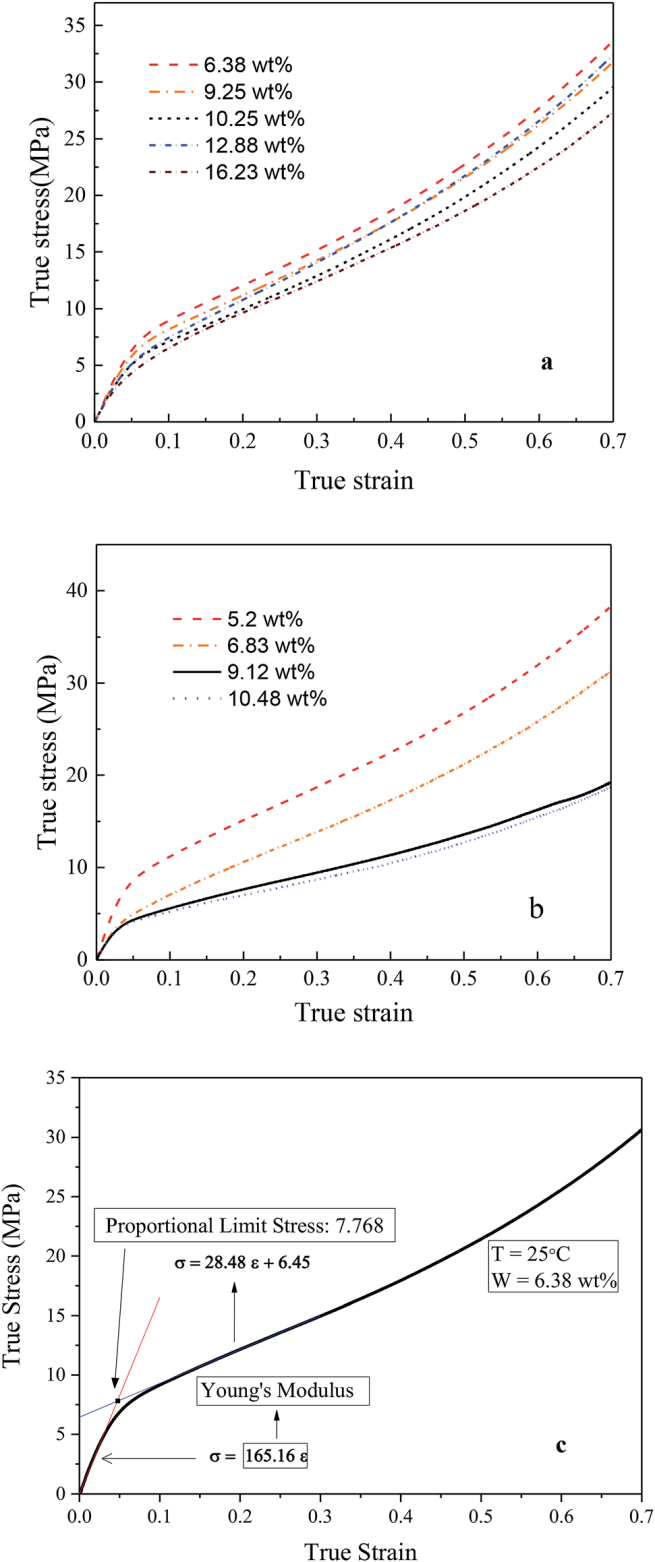
True stress–true strain curves of (a) Nafion® 117 and (b) Nafion® 212 at different water contents, (c) determination of yield point.

Fig. 4a and b demonstrated the typical TS–TS curves of Nafion® 117 and 212 membranes, respectively. It is the characteristics of semicrystalline polymers that a small elastic region (strain less than yield strain) followed by gradual rollover yield and post-yield strain hardening periods. In the post-yield region, the strain-hardening slope increased slightly with the increasing strains. The initial slopes of TS–TS curves were taken as the Young's moduli of hydrated membranes. The Young's moduli were calculated according to the linear fitting (with an error of 0.3%); the critical linear-elastic strains (*ε*_c_), the largest linear elastic strain, were then obtained (Table 1). The proportional limit stress (PLS), which can be defined graphically as the stress at the intersection of tangents to the initial linear portion of TS–TS curve and the initial strain hardening response, was used to identify the onset of yield limit (plastic deformation) (Fig. 4c), and the calculated yield stresses and strains are presented in Table 1. It was noticed that for both membranes, Young's modulus, critical elastic strain, and yield stress/strain all demonstrated a decreasing trend with the increasing humidity. At the same humidity, Nafion® 117 manifested larger linear-elastic region in comparison to Nafion® 212, whereas Nafion® 212 yielded higher Young's modulus as compared to Nafion 117, thus indicating that Nafion® 212 is stiffer than Nafion® 117. The changes in TS–TS curves of Nafion® 117 were not clear in the humidity range of 6–16 wt%, and this was also true for Nafion® 212 for humidity greater than 9 wt%. At smaller humidity, a decreasing trend was noticed in the initial slopes of TS–TS curves.^14–16^

**Table 1:** Membrane characteristics at different water contents

Water content (wt%)	Young's modulus (MPa)	*ε*_c_	Yield stress (MPa)	Yield strain (MPa)
**Nafion 117**				
6.38	165.16	0.03	7.77	0.047
9.25	127.55	0.041	6.79	0.056
10.25	116.79	0.032	5.77	0.052
12.88	115.42	0.034	5.9	0.053
16.23	109.96	0.023	5.38	0.053

**Nafion 212**				
5.20	228	0.024	8.95	0.04
6.83	144	0.018	4.88	0.037
9.12	133	0.017	4.24	0.037
10.48	130	0.017	4.04	0.036

The TS–TS curves can be explained by the swelling characteristics and deformation mechanism of Nafion membranes. It is well known that the deformation of membranes at mesoscale can be described in terms of the rotation of bundles comprised of rod-like polymer aggregates (at low strains) and the subsequent orientation and elongation of polymer-chains (at high strains),^47,48^ which roughly correspond to elastic and plastic deformation, respectively. The higher the degree of winding and aggregation of polymer chains, the longer the elastic strain region will be. Therefore, Nafion® 212, with a lower winding degree of polymer chains (indicated by small values of *V*_w–s_), had a smaller elastic region than Nafion® 117 at the same humidity. This inference was consistent with the TS–TS curve (Fig. 4) and Table 1. Besides, because the two membranes have different thicknesses and also number of polymer chains, the yield deformation region (strain ranging from yield strain to failure strain) cannot be further estimated.

### 4.2 Nano-mechanical behavior of hydrated PFSA ionomers

#### 4.2.1 Swelling behavior

**Fig. 5:**
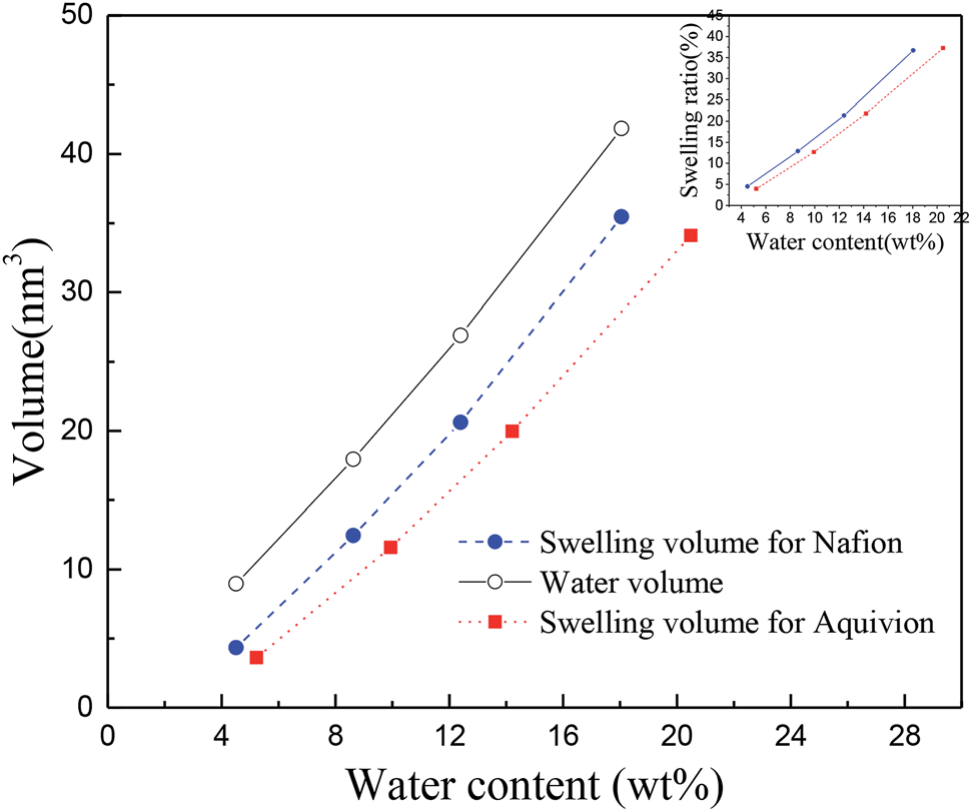
Volume-water content curve for nanoscale parts of Nafion and Aquivion membranes. The inset figure shows the relationship of swelling ratio and water content (blue color and red color are for Nafion and Aquivion, respectively).

**Fig. 6:**
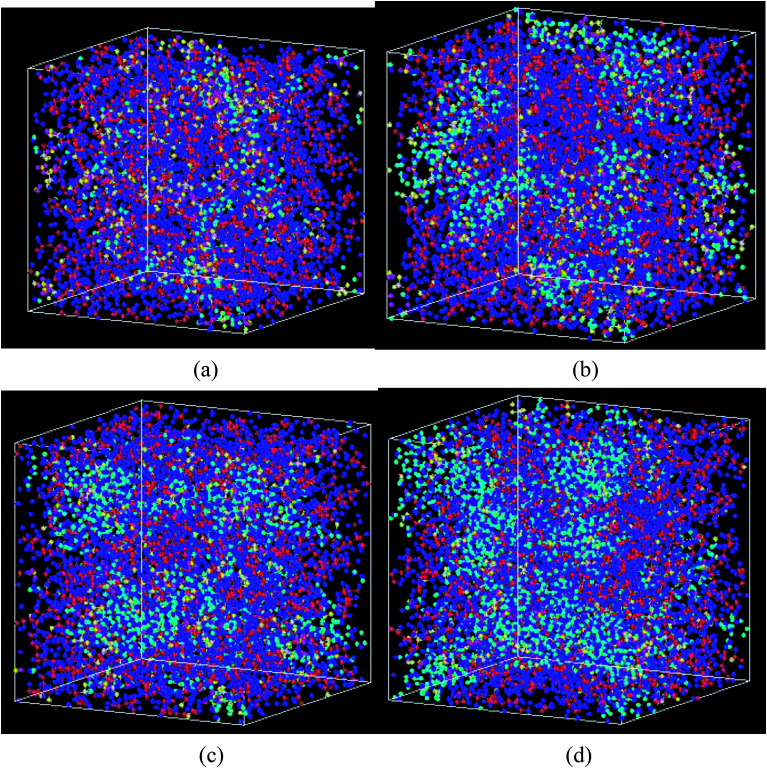
The swelling structure of Nafion polymer at a hydration level of (a) *λ* = 3 (b) *λ* = 6 (c) *λ* = 9, and (d) *λ* = 14 (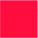
C, 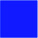
F, 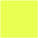
O (between C atoms in side chain), 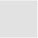
S, 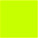
O (SO_3_^−^), 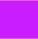
H(H_3_O^+^), 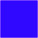
O(H_2_O), 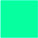
H(H_2_O)).

Similar with Nafion bulk membrane, the swelling volume of nanoscale ionomer was also smaller than the volume of the uptake water (Fig. 5), demonstrating the entanglement characteristics among polymer chains. Moreover, Nafion ionomers exhibited the lower values of *V*_w–s_ than Aquivion ones at the same water content, showing that long side chain can reduce the winding degree. However, the values of *V*_w–s_ in nanoscale were highly small (in the order of nm^3^), demonstrating a more uniform dispersion of polymer chains (Fig. 6a) as compared to bulk membrane. The swelling ratios of two types of ionomers were both higher than those of bulk membrane. Moreover, Nafion with long side chain had a higher swelling ratio than Aquivion membrane at the same water content. This result was agreed with the literature^39^ that the nanoscale film exhibited reduced swelling but higher swelling ratio due to the thinner thickness and more uniform distribution of polymer chains (dispersion of polymer chains at molecular level).

Fig. 6 shows the structural changes of Nafion ionomer during swelling, showing that extruded water molecules expand the distance between polymer chains. With the increasing of hydration level, the distance between polymer chains is enhanced, and water connectivity gradually forms and enlarges. However, the entanglement and aggregation between polymer chains are still visible at the hydration level of 14.

#### 4.2.2 True stress–true strain curves

##### 4.2.2.1 Effects of side chain and hydration level

**Fig. 7:**
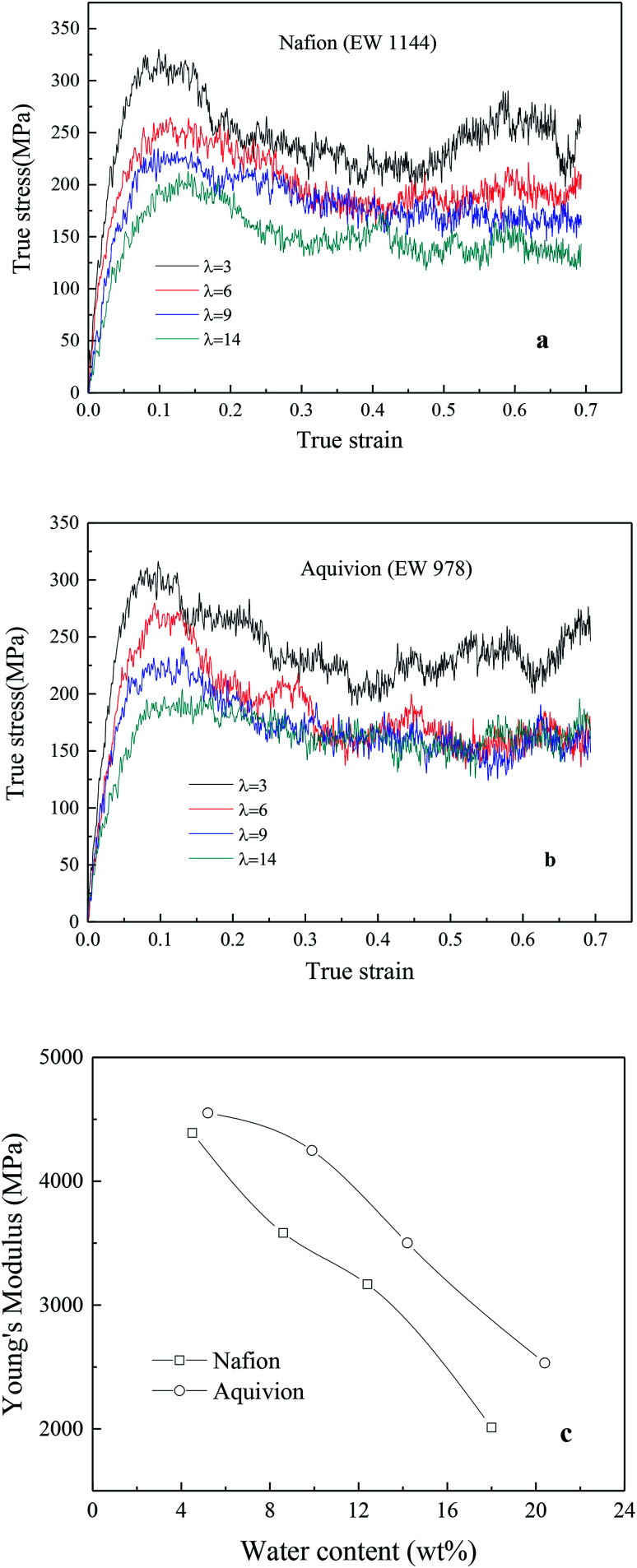
True stress–true strain curves of (a) Nafion and (b) Aquivion membrane, (c) relationship between Young's modulus and water content.

Fig. 7a and b exhibited the TS–TS curves of Nafion and Aquivion for four different hydrated levels (water contents for Nafion were 4.5 wt% (*λ* = 3), 8.6 wt% (*λ* = 6), 12.4 wt% (*λ* = 9), and 18 wt% (*λ* = 14); water contents for Aquivion were 5.2 wt% (*λ* = 3), 10 wt% (*λ* = 6), 14.2 wt% (*λ* = 9), and 20.4 wt% (*λ* = 14)). The elastic region was observed at strains less than 0.05 around followed by a gradual rollover yield period in the strain range of 0.05–0.15. Subsequently, a conspicuous decrease in stress was noticed, and finally, a relatively steady period was obtained for true strains less than or equal to 0.7. It was worth noting that no strain-hardening region was observed. This was because that the sliding and disentangling of a few polymer chains in nanoscale ionomers occurred easily at high strains during stretching, and the appearance of pinhole caused the decrease in stress during further stretching.

In addition, the initial slopes of TS–TS curves decreased with the increasing hydrated levels (*λ*) due to polymeric aggregation at lower strains, this trend was similar to bulk membranes. However, it is known that nanoscale polymers manifest higher modulus with more anisotropy. Moreover, the adopted large strain rate (10^9^ s^−1^) is also the main reason for obtaining higher Young's modulus and yield stress from simulation as compared to macro-mechanical testing.^31,33^ The Young's moduli of Aquivion and Nafion membranes were displayed as the function of water content in Fig. 6c. Higher Young's moduli were obtained from Aquivion membrane as compared to Nafion; this conclusion was consistent with that of many simulation literatures that shorter side chains can increase the values of Young's modulus.

##### 4.2.2.2 Effects of backbone length and strain rate

**Fig. 8:**
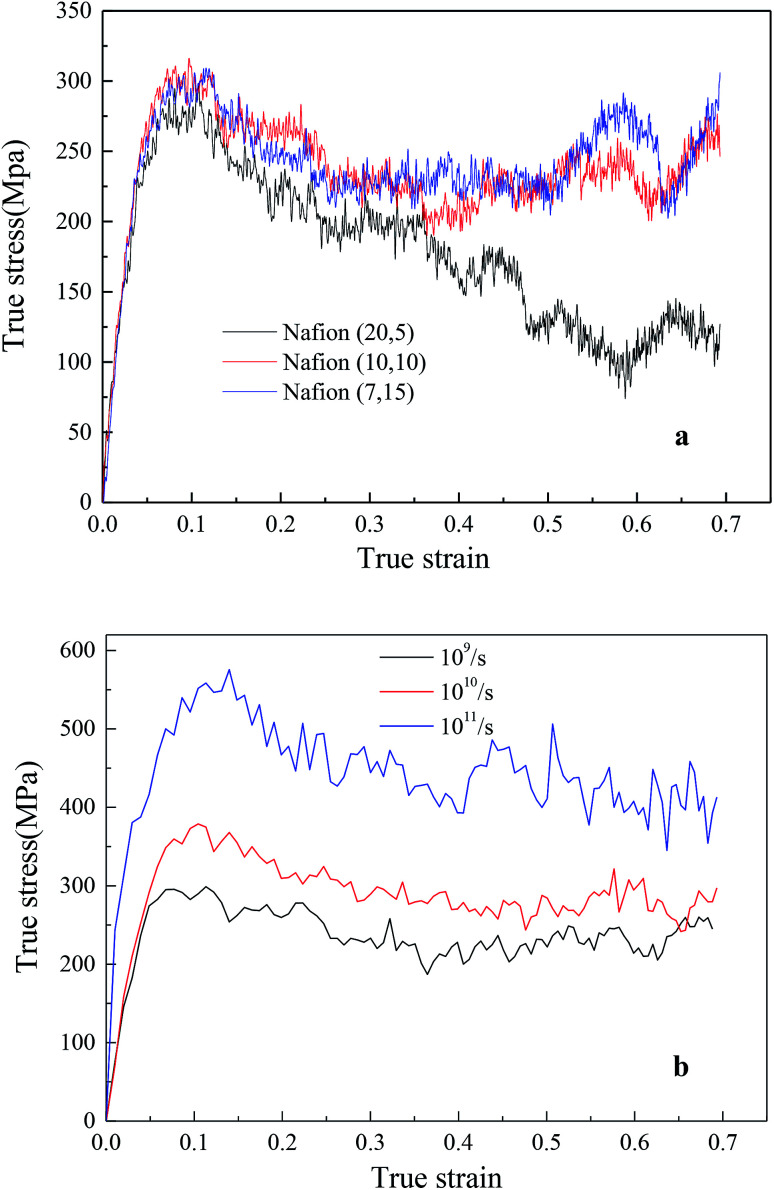
True stress–true strain curves of hydrated Nafion at *λ* = 3: (a) with different number of monomers in a chain and (b) at different strain rates. Nafion (20, 5) denotes that there are 20 chains and 5 monomers in each chain.

The effects of different monomer in each chain (5, 10 and 15) of hydrated Nafion (*λ* = 3) were investigated, as shown in Fig. 8a. No obvious difference was observed in the elastic region of TS–TS curves, thus demonstrating that backbone length had little effect on elastic modulus. However, as the system with the shortest backbone length (five monomers in a chain) had the smallest interaction force (van der Waal's force) between chains, the sliding and disentangling of polymer aggregates easily occurred in the failure process, showing a marked decline trend in the strain range of 0.2–0.7. Further, for 10 or more than 10 monomers in a chain, backbone length yielded a small impact on both plastic and failure deformation. In addition, the effects of strain rate on hydrated Nafion (10, 10) were investigated. It was observed that mechanical responses of Nafion were more sensitive to strain rate in comparison to humidity and backbone length (Fig. 8b).

#### 4.2.3 The deformation mechanism of PFSA ionomers

**Fig. 9:**
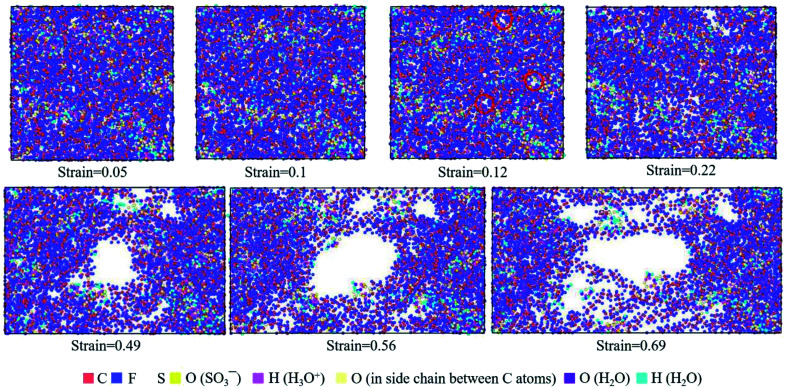
Top view of molecular structures of hydrated Nafion (*λ* = 3) at different true strains in the *xz* plane.

The deformation mechanism of hydrated Nafion at *λ* = 3 can be well described by the molecular structures at various strains. Fig. 9 revealed that when the value of true strain was less than 0.1, a clear phase separation was observed, and various nanoparticles including polymer chains, water molecules, and hydronium ions were distributed evenly in the molecular structure. When the value of strain was larger than 0.12, the sliding and disentangling of polymer aggregates began from the regions of water molecules, and consequently, small holes were formed (red circles in Fig. 9), thus causing a gradual decrease in stress (Fig. 7a). At higher strain (greater than 0.3), polymer chains moved with preferential orientation along the tensile direction, and consequently, the structure became loose and larger holes appeared. Hence, only a few chains were left to supply the successive deformation; however, the rupture of polymer structure did not occur until the true strain reached 0.69.

**Fig. 10:**
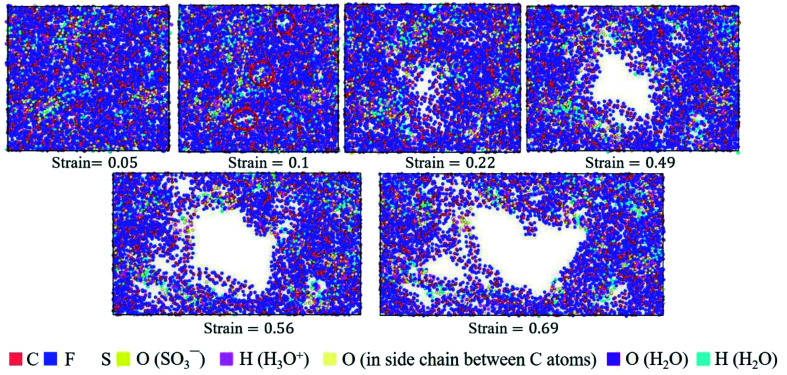
Top view of molecular structures of hydrated Aquivion membrane (*λ* = 3) at different true strains in the *xz* plane.

The hydrated Aquivion ionomers had the similar deformation process as Nafion ones. It was depicted in Fig. 10 that for the Aquivion ionomer at *λ* = 3 the failure of small holes occurred at the true strain of 0.1, which was slightly smaller than that of Nafion. It was consistent with our result that low value of *V*_w–s_ reflected a small elastic–plastic region (see Fig. 7a and b), where the ionomers should have the similar scales. Furthermore, with the increasing of strain, larger holes appeared and some chains were still left to resist the tensile deformation. Therefore, the damage of ionic polymers was mainly caused by chain slip rather than bond breakage.

## 5. Conclusions

In the afore-discussed research, mechanical behavior of hydrated PFSA membrane at mesoscale and nanoscale were investigated. The volume of uptake water was larger than swelling volume for PFSA membranes at mesoscale and nanoscale, demonstrating the entanglement characteristics of polymer chains. At the same humidity, the value of *V*_w–s_ of Nafion® 117 was highly larger than that of Nafion® 212, reflecting a higher polymeric aggregation and larger yield strain. Nano-scale Nafion ionomer had a greater value of *V*_w–s_ and larger elastic–plastic region than Aquivion one. For the nanoscale PFSA ionomers with the same backbone, long side chain can reduce the interface force among polymer chains and increase their dispersion degree. In the TS–TS curves of hydrated PFSA ionomers at nano scale, no strain-hardening region was found. This is due to the fact that the polymer chains were few and evenly dispersed, thus the ability to resist deformation was greatly weakened in the plastic deformation stage as compared to bulk membrane. The method for analyzing the correlation of swelling and mechanical behavior of PFSA membrane may be applicable to most of polymers.

## Conflicts of interest

There are no conflicts to declare.

## Supplementary Material

RA-009-C9RA00745H-s001
