# Supplementary material for: Mechanical behavior of a hydrated perfluorosulfonic acid membrane at meso and nano scales
Source: RSC Adv. 2019 Mar 26;9(17):9594–603. doi: 10.1039/c9ra00745h (PMC9062152; doi:10.1039/c9ra00745h)
Supplement: RA-009-C9RA00745H-s001 [file RA-009-C9RA00745H-s001.pdf]

Electronic Supplementary Material (ESI) for RSC Advances.
This journal is © The Royal Society of Chemistry 2019

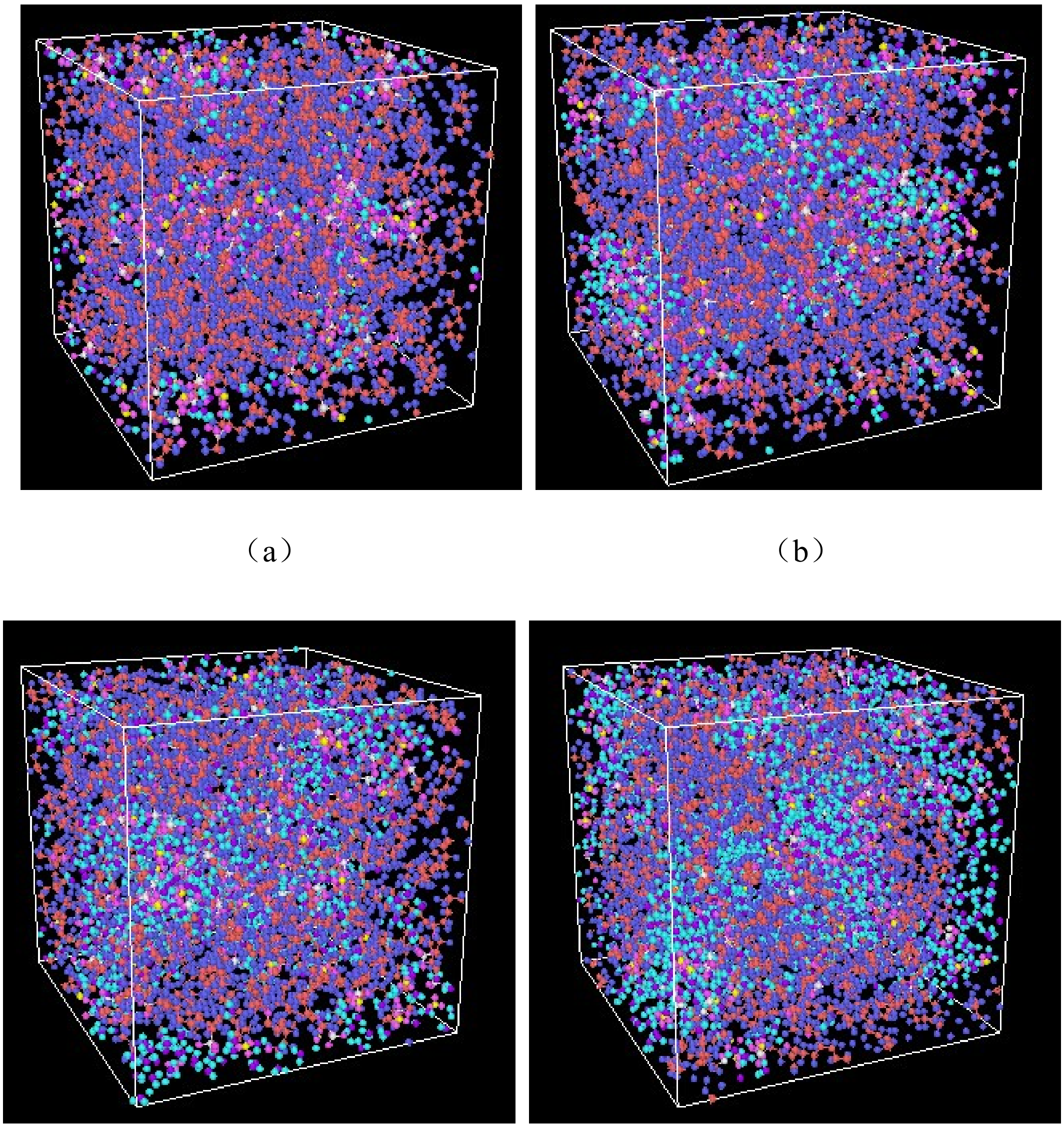

(a) (b)

(c) (d)

Figure The swelling structure of Aquivion polymer at a hydration level of (a) λ=3 (b) λ=6 (c) λ=9, and (d) λ=14 (C, F, O (between C atoms in side chain), S, O (SO_3^-), H(H_3O^+), O(H_2O), H(H_2O)).

The results of this paper can provide a mechanism explanation for swelling and mechanical behavior of PFSA polymer membranes
